# Ventilation Prior to Umbilical Cord Clamping Improves Cardiovascular Stability and Oxygenation in Preterm Lambs After Exposure to Intrauterine Inflammation

**DOI:** 10.3389/fped.2018.00286

**Published:** 2018-10-25

**Authors:** Alessandra Lio, Claudia Aurilia, Valerie Zahra, Timothy J. Moss, Domenic A. LaRosa, Stuart B. Hooper, Andrew W. Gill, Martin Kluckow, Ilias Nitsos, Giovanni Vento, Graeme R. Polglase

**Affiliations:** ^1^Division of Neonatology, Fondazione Policlinico Universitario A. Gemelli IRCCS, Rome, Italy; ^2^Department of Obstetrics and Gynaecology, The Ritchie Centre, Monash University and Hudson Institute of Medical Research, Melbourne, VIC, Australia; ^3^Centre for Neonatal Research and Education, University of Western Australia, Perth, WA, Australia; ^4^Department of Neonatology, Royal North Shore Hospital and University of Sydney, Sydney, NSW, Australia; ^5^Division of Neonatology, Universita Cattolica del Sacro Cuore, Rome, Italy

**Keywords:** delayed cord clamping, premature birth, chorioamnionitis, placental transfusion, physiological-based cord clamping

## Abstract

**Background:** Delaying umbilical cord clamping until after aeration of the lung (physiological-based cord clamping; PBCC) maintains cardiac output and oxygenation in preterm lambs at birth, however, its efficacy after intrauterine inflammation is not known. Given the high incidence of chorioamnionitis in preterm infants, we investigated whether PBCC conferred any benefits compared to immediate cord clamping (ICC) in preterm lambs exposed antenatally to 7 days of intrauterine inflammation.

**Methods:** Ultrasound guided intraamniotic injection of 20 mg Lipopolysaccharide (from *E. coli*:055:B5) was administered to pregnant ewes at 0.8 gestation. Seven days later, ewes were anesthetized, preterm fetuses exteriorised via cesarean section, and instrumented for continuous measurement of pulmonary, systemic and cerebral pressures and flows, and systemic, and cerebral oxygenation. Lambs were then randomized to either PBCC, whereupon ventilation was initiated and maintained for 3 min prior to umbilical cord clamping, or ICC where the umbilical cord was cut and ventilation initiated 30 s later. Ventilation was maintained for 30 min.

**Results:** ICC caused a rapid fall in systemic (by 25%) and cerebral (by 11%) oxygen saturation in ICC lambs, concurrent with a rapid increase in carotid arterial pressure and heart rate. The overshoot in carotid arterial pressure was sustained in ICC lambs for the first 20 min of the study. PBCC maintained cardiac output and prevented the fall in cerebral oxygen delivery at birth. PBCC lambs had lower respiratory compliance and higher respiratory requirements throughout the study.

**Conclusion:** PBCC mitigated the adverse effects of ICC on oxygenation and cardiac output, and therefore could be more beneficial in preterm babies exposed to antenatal inflammation as it maintains cardiac output and oxygen delivery. The increased respiratory requirements require further investigation in this sub-group of preterm infants.

## Introduction

Preterm birth is still the commonest cause of neonatal death and morbidity worldwide (1). Preterm infants often require extensive immediate postnatal care. They are at risk of respiratory and cardiovascular diseases and more likely to have neurodevelopmental disability (1) then their term counterparts. Intrauterine inflammation is implicated in the majority of extremely preterm births (2). The majority of fetuses exposed to chorioamnionitis develop a systemic inflammatory response [the fetal inflammatory response syndrome; FIRS, (3)] which has several consequences for development of the fetal and neonatal cardiopulmonary, cerebral, and renal systems (4), and can alter the cardiovascular transition at birth (5).

Delayed umbilical cord clamping (DCC) improves hemodynamic stability at birth, decreases the need of blood transfusion, the rate for necrotising enterocolitis, and peri/intraventricular hemorrhage (P/IVH) (6). The benefits of DCC have been attributed to net placenta-fetal blood transfusion and an increase in neonatal blood volume (7, 8) but we have previously demonstrated that the maintenance of cardiac output throughout the transition at birth may be a more significant benefit (9). The establishment of breathing/aeration of the lungs prior to cord clamping, termed physiological-based cord clamping (PBCC), may be more important than the delay in cord clamping itself (9). Initiation of ventilation prior to cord clamping in preterm lambs increases pulmonary blood flow (PBF) before the cord is clamped, allowing for the pulmonary circulation to sustain left ventricular filling and hence cardiac output upon cord clamping, and establishing the role of pulmonary gas-exchange from the placenta (9). PBCC in preterm lambs improves cardiovascular and cerebral circulatory stability and adequate oxygenation throughout delivery (10). However, the influence of intrauterine inflammation on this process is unknown.

Chorioamnionitis is an independent risk factor for cerebral palsy (11–13). It is also an independent risk factor for P/IVH (14) and for toddler behavior that is later associated with autism disorders (15) among preterm babies. Antenatal inflammation can cause systemic hemodynamic impairment, cerebral blood flow instability and loss of cerebral autoregulation, which are crucial mechanisms involved in the development of brain injury (16, 17). Our previous studies found increased carotid blood pressure and flow in preterm lambs 2, 4, and 7 days after lipopolyxaccharide (LPS) exposure (18) indicating a high cerebral oxygen consumption and metabolic demand following exposure to intrauterine inflammation. Antenatal inflammation exposure also has significant adverse effects on fetal pulmonary vascular development (19, 20) and the heart structure of preterm lambs in terms of heart growth, contractile function and enhanced vulnerability to reperfusion injury and stress (21). These alterations to the cardio-pulmonary vasculature are associated with an increase in pulmonary arterial pressure and vascular resistance resulting in increased postnatal right-to-left shunting through the ductus arteriosus, with subsequent cardiovascular sequelae, including decreased left ventricular output (LVO) (22). Moreover, preterm lambs exposed to a single injection of intra-amniotic LPS 7 days prior to delivery had a slower rate of increase in mean, end-systolic and end-diastolic pulmonary blood flow (PBF) and LVO immediately after birth, suggesting that the cardiopulmonary hemodynamic transition is impaired in LPS-exposed lambs immediately after delivery (5).

Given that intrauterine inflammation has significant consequences on the pulmonary, cardiovascular and cerebral systems, which result in an altered cardiovascular transition at birth, the aim of this study was to determine whether PBCC can improve the cardiovascular transition at birth in preterm lambs exposed to intrauterine inflammation. We hypothesized that PBCC will improve cardiovascular stability and cerebral oxygenation following preterm birth compared to immediate cord clamping in lambs exposed to antenatal LPS for 7 days.

## Methods

### Ethics

The experimental protocol was performed in accordance with guidelines established by the National Health and Medical Research Council of Australia and was approved by the Monash Medical Centre (MMCA) animal ethics committee at Monash University.

### Experimental protocol

At 118 ± 2 (mean ± standard deviation) days of gestation (term~147 days), pregnant ewes bearing singleton fetuses received an ultrasound-guided injection of LPS (*Escherichia coli* 055:B5, 20 mg; Sigma Aldrich, NSW, Australia) into the amniotic sac. Confirmation that injections were given into the amniotic sac (and not the allantois) was verified by subsequent electrolyte analysis of aspirated fluid (23).

Seven days after intra-amniotic LPS, lambs were delivered by cesarean section under general anesthesia. The ewes were anesthetized with an intravenous bolus of 5% sodium thiopentone (Pentothal; 1 g in 20 ml) and, following intubation, maintained with inhalational anesthesia (isoflourane 1.5–2.5% in 70/30 O_2_/N_2_O). The fetal head, neck, and chest were exposed via cesarean section and vascular flow probes of appropriate size (Transonic Systems, Ithaca, NY, USA) were placed around the left main pulmonary artery and left carotid artery for monitoring of PBF and carotid arterial blood flows (CaBF), respectively. Carotid arterial blood flow is related to total brain blood flow in sheep (24). Heparinised saline-filled polyvinyl catheters were inserted into the other carotid artery and into a jugular vein.

A transcutaneous arterial oxygen saturation (SpO_2_) probe (Masimo, Radical 4, CA, USA) was placed around the right forelimb and the output recorded continuously. Near Infrared Spectroscopy optodes (Casmed Foresight, CAS Medical Systems Inc, Branford, CT, USA) were placed over the left frontal cortex and on the right leg used to continuously measure cerebral (SctO_2_) and peripheral tissue oxygen saturation, respectively. The fetal trachea was intubated with a clamped, 4.0 mm cuffed endotracheal tube. After completion of instrumentation, the ewe was rotated onto its side and the fetus was completely exteriorized from the uterus, still attached to the umbilical cord. The fetus was dried, placed on a delivery table immediately next to the ewe and covered with a plastic bag. A rectal thermometer was used to ensure that lambs were kept in a neutral thermal environment. The lung liquid was drained passively for 10 s or until liquid ceased exiting the airways. Chorioamnion tissue was sampled for assessment of inflammation using CD45 immunohistochemistry. Each fetus was randomized to either umbilical cord clamping prior to initiation of ventilation (ICC; *n* = 6) or the initiation of ventilation prior to umbilical cord clamping (PBCC; *n* = 6) groups. In ICC lambs, the umbilical cord was immediately clamped and cut, and ventilation commenced 30 s later. In PBCC lambs, ventilation commenced while the umbilical cord remained patent, and umbilical cord clamping was performed 3 min after ventilation onset. Once stabilized, the lamb was transferred to an infant warmer (CosyCot, Fisher and Paykel, Auckland, New Zealand) to avoid hypothermia.

In both groups ventilation was initiated with a tidal volume of 7 mL/kg and a positive end-expiratory pressure of 5 cmH_2_O using warmed and humidified inspired gases, with an initial fraction of inspired oxygen (FiO_2_) of 21% (Babylog 8000+, Dräger, Lübeck, Germany). Peak inspiratory pressure was limited to 40 cmH_2_O to avoid pneumothoraces. During the ventilation period regular blood-gas analysis (ABL30, Radiometer, Copenhagen, Denmark) was performed on simultaneous arterial and venous blood samples. FiO_2_ was adjusted to target SpO_2_ of 93–98% and PaO_2_ 60–100 mmHg and ventilation to target PaCO_2_ between 40 and 60 mmHg. All lambs received sedation (Alfaxane i.v. 5–15 mg/kg/h in glucose; Jurox, East Tamaki, Auckland, New Zealand) to prevent spontaneous breathing during the experiment. The ewes were humanely killed using sodium pentobarbitone (100 mg/kg i.v) after cesarean section without recovery from anesthesia. Lambs were euthanized after completion of the 30 min ventilation study by the same method.

### Measurements

Instantaneous blood flow in the carotid artery was used as a proxy for cerebral blood flow (CBF) (26), SaO_2_ and SctO_2_ were recorded digitally using a data acquisition system (Powerlab; ADInstruments, Castle Hill, Australia). Arterial pressures were measured using pressure transducers (PD10; DTX Plus Transducer; Becton Dickinson, Singapore) and also recorded digitally.

### Calculations

Specific dynamic lung compliance (Cdyn,spec) = V_T_ (ml.kg^−1^ birth-weight)/ΔP (cmH_2_O), where ΔP = (PIP-PEEP).

Ventilation efficiency index (VEI) = 3800/ΔP × f × PaCO2, where 3800 = CO_2_ production constant and f = breathing frequency (25).

The alveolar arterial difference in oxygen (AaDO_2_) was calculated as described previously (26).

Cerebral oxygen delivery, arterial and venous oxygen content, cerebral oxygen extraction, and consumption were calculated as described previously (27).

### Statistics

Data are presented as mean ± SD. The sample size was calculated to be sufficient to detect a 30% alteration in transitional physiology (pulmonary and cerebral blood flows and pressures) using 2-way repeated measures ANOVA (power of 0.8 and *p* < 0.05) as demonstrated previously (9). This information has been added to the methods. Fetal data collected prior to intervention were compared using Students *t*-test. All subsequent physiological data were compared over time and between groups using a two-way repeated measures ANOVA with *post hoc* analysis (Holm-Sidak) determining the time that differences were evident (Sigmastat v3.0, SPSS Inc.). In ICC lambs time zero was set at cord clamping, while in PBCC time zero was set at initiation of ventilation. Statistical significance was accepted for *p* < 0.05.

## Results

### Baseline characteristics

All injections were confirmed to be into the amniotic sac. The presence of intrauterine inflammation was confirmed by the presence of CD45+ inflammatory cell infiltration in the fetal membranes, and was not different between groups (PBCC: 54.1 ± 16.8 vs. ICC: 46.7 ± 10.1). Fetal body weights, sex, and arterial blood gas parameters were similar between groups prior to delivery (Table 1).

**Table 1:** Fetal characteristics.

	**ICC**	**PBCC**
*N* (males)	6 (1)	6 (1)
Weight (kg)	3.19 ± 0.45	3.24 ± 0.34
pH	7.30 ± 0.03	7.27 ± 0.04
paO_2_ (mmHg)	21.6 ± 4.8	23.8 ± 8.7
paCO_2_ (mmHg)	47.3 ± 3.9	53.0 ± 9.7
SaO_2_ (%)	55.9 ± 12.9	60.0 ± 22.7
Hb (g/dl)	10.3 ± 1.1	9.9 ± 1.6

### Respiratory parameters and oxygenation

**Figure 1:**
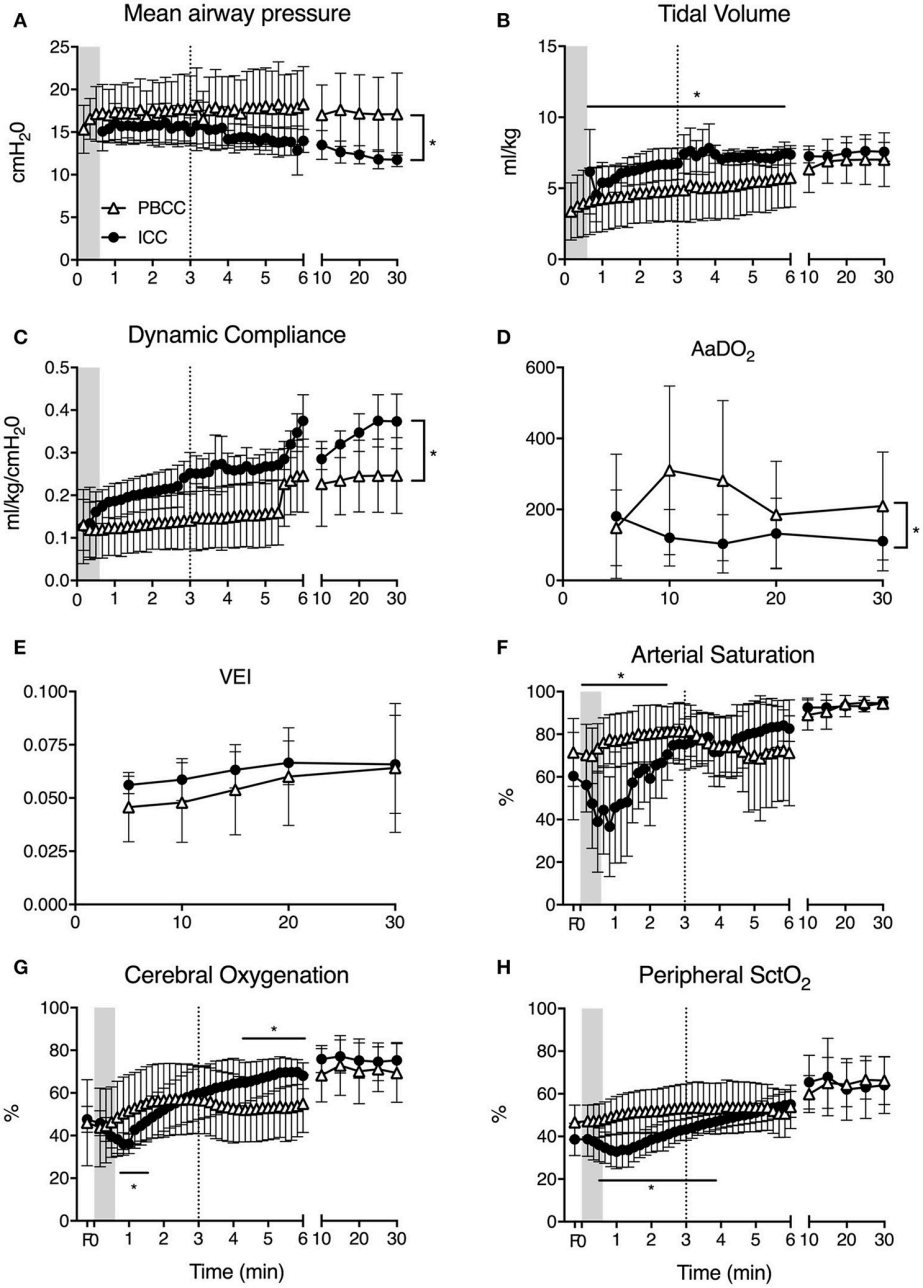
**(A)** Mean airway pressure, **(B)** tidal volume, **(C)** respiratory dynamic compliance, **(D)** alveolar-arterial difference in oxygen (AaDO_2_), **(E)** ventilator efficiency index (VEI), **(F)** arterial saturation (SaO_2_), **(G)** cerebral, and **(H)** peripheral tissue oxygenation (SctO_2_) measured in PBCC (open triangles) or ICC (closed circles) preterm lambs exposed to antenatal LPS for 7 days. Shaded region indicates the time between cord clamping and ventilation onset in ICC lambs. Dashed line represents time of cord clamping in PBCC lambs. *indicates significant difference between groups.

Peak inflation pressures and mean airway pressures (Figure 1A) were significantly higher in PBCC lambs throughout the study and tidal volumes (Figure 1B) were significantly lower for the first 6 min compared to ICC lambs. Indeed, PBCC lambs were unable to obtain target tidal volumes in the first 5 min when using a maximum peak inflation pressures of 40 cmH_2_O. Dynamic compliance (Figure 1C) was significantly lower in PBCC lambs. Further, AaDO_2_ (Figure 1D) was significantly higher in the PBCC lambs than ICC lambs, indicative of worse oxygenation in the PBCC lambs. A higher fraction of inspired oxygen requirement drove this. In spite of worse respiratory status, minute ventilation, PaCO_2_, PaO_2_, and ventilator efficiency index (Figure 1F) were not different between groups, indicating appropriate ventilator management. Lactate levels were significantly higher at 5 and 10 min in ICC lambs compared to PBCC (5 min: PBCC 3.6 ± 0.9 vs. ICC 5.1 ± 0.9 mmol/l; 10 min: PBCC 3.6 ± 0.9 vs. ICC 5.6 ± 1.6 mmol/l), but not thereafter.

### Oxygenation

Clamping of the umbilical cord prior to ventilation resulted in a decrease in arterial oxygen saturation (Figure 1F). In the time taken between cord clamping and ventilation onset in the ICC group, SaO_2_ fell from 57 to 32%. Cerebral and peripheral SctO_2_ similarly fell during this time (Figures 1G,H). Initiation of ventilation in the ICC group led to an increase in arterial saturation, cerebral and peripheral SctO_2_ that reached values similar to PBCC lambs in 2.5, 1.5, and 4 min, respectively. In PBCC lambs there was a steady increase in SaO_2_, cerebral and peripheral SctO_2_ after ventilation start. Subsequent clamping of the umbilical cord saw a fall in arterial oxygen saturation by ~7% over the next 3 min, however no difference was observed between groups during this time, and the remaining 25 min of the study. Cerebral oxygen consumption, oxygen delivery, and oxygen extraction were not different between groups at any time point measured (data not shown).

### Haemodynamic parameters

**Figure 2:**
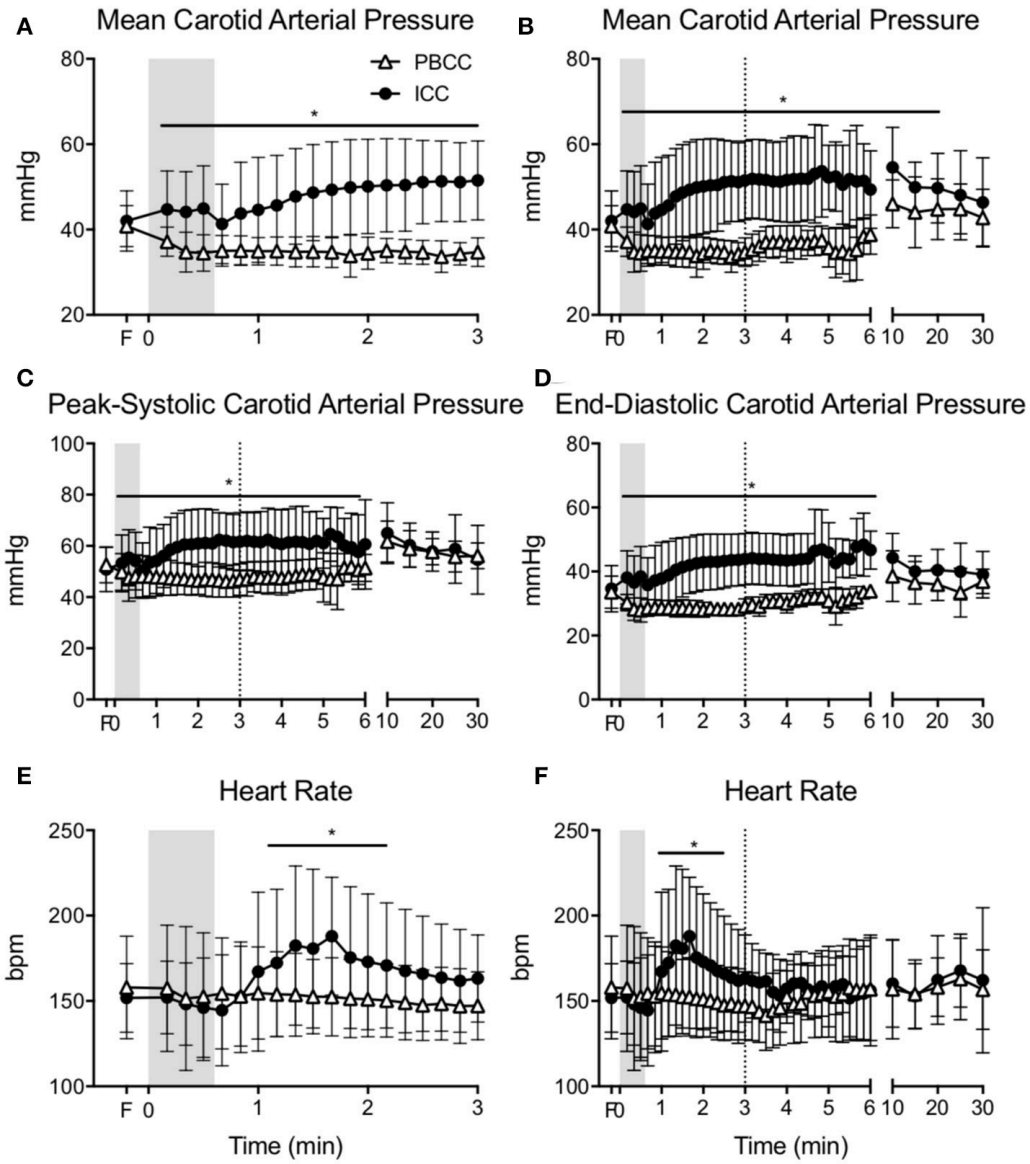
**(A)** Mean carotid arterial pressure during the first 3 min and **(B)** throughout the study, **(C)** peak-systolic, and **(D)** end-diastolic carotid arterial pressure, **(E)** heart during the first 3 min, and **(F)** heart rate throughout the study measured in PBCC (open triangles) or ICC (closed circles) preterm lambs exposed to antenatal LPS for 7 days. Shaded region indicates the time between cord clamping and ventilation onset in ICC lambs. Dashed line represents time of cord clamping in PBCC lambs. *indicates significant difference between groups.

Carotid arterial pressure significantly increased upon cord clamping in ICC lambs (Figure 2A). Upon ventilation onset mean arterial pressure continued to increase and remained higher for the first 20 min (Figure 2B). Systolic and diastolic arterial pressure was significantly higher in ICC lambs compared to PBCC for the first 10 min after birth (Figures 2C,D). Conversely, mean, systolic, and diastolic pressures decreased after ventilation onset in PBCC lambs (although not significantly) and increased progressively upon cord clamping. Heart rate rapidly increased after ventilation onset in ICC lambs and was significantly higher than PBCC between 1 and 2 min (Figure 2E). Heart rate was not different between groups thereafter (Figure 2F).

**Figure 3:**
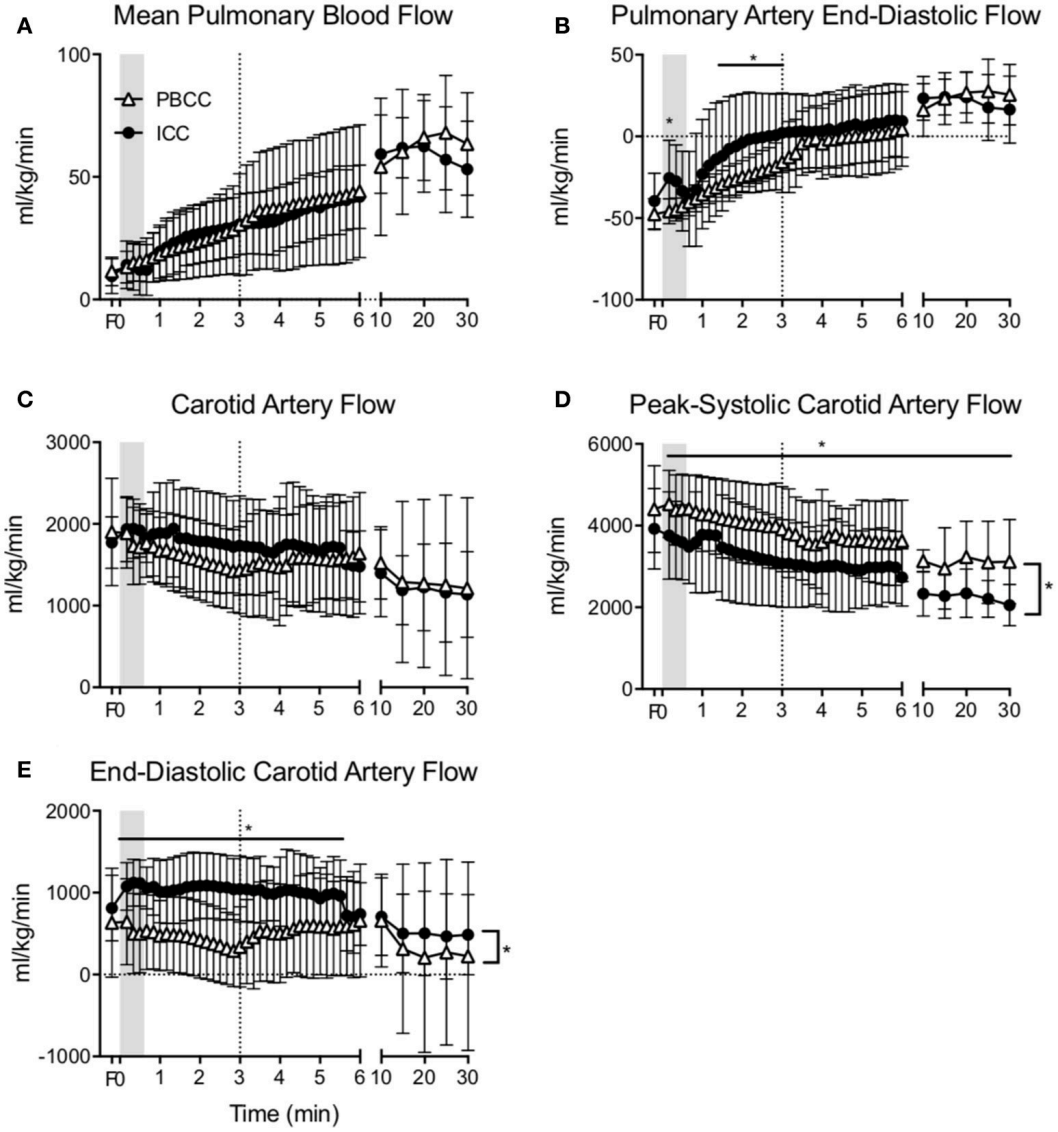
**(A)** Mean pulmonary blood flow, **(B)** end-diastolic pulmonary blood flow, **(C)** mean carotid arterial flow, **(D)** peak-systolic, and **(E)** end-diastolic carotid arterial flow measured in PBCC (open triangles) or ICC (closed circles) preterm lambs exposed to antenatal LPS for 7 days. Shaded region indicates the time between cord clamping and ventilation onset in ICC lambs. Dashed line represents time of cord clamping in PBCC lambs. *indicates significant difference between groups.

Mean pulmonary blood flow increased upon ventilation onset similarly in both groups (Figure 3A) and no difference was observed throughout the study. End-diastolic pulmonary blood flow was lower in PBCC compared to ICC lambs upon ventilation onset but this difference was lost at 3 min (Figure 3B).

Mean carotid blood flow in ICC lambs followed the changes in arterial pressure during the first minute after birth; an initial increase was observed upon cord clamping followed by a decrease upon ventilation onset (Figure 3C). Mean carotid blood flow in PBCC decreased upon ventilation onset. Overall mean carotid arterial blood flow was not different between groups. However, peak-systolic carotid arterial blood flow (Figure 3D) was significantly higher, and end-diastolic flow (Figure 3E) was significantly lower in PBCC lambs compared to ICC, resulting in a higher cerebral pulsatility index (data not shown).

## Discussion

A stable transition at birth is crucial for ensuring neonatal well-being and survival. Delaying umbilical cord clamping until after aeration of the lung (PBCC) in preterm lambs markedly improves cardiovascular function and stabilizes cerebral hemodynamics and oxygenation (9, 10, 28). Several clinical trials have examined the effects of delayed cord clamping in preterm infants who need resuscitation at birth [e.g., (7, 29)], however, sub-group analysis of vulnerable infants, such as those exposed to antenatal inflammation, has not been done (30). This is a crucial problem since, in most cases, extreme prematurity is associated with chorioamnionitis and intrauterine inflammation (2, 4). Fetuses exposed to chorioamnionitis often develop a fetal inflammatory response syndrome (3), which has several consequences on neonatal cardiopulmonary and cerebral systems (5). In this study we compared PBCC to ICC in preterm lambs exposure to LPS 7 days prior to delivery. We observed rapid falls in systemic and cerebral oxygenation in ICC lambs concurrent with rapid fluctuations in carotid arterial pressure and flow and heart rate. These changes were not present in PBCC lambs suggesting improved cardiovascular stability and maintenance of oxygenation in these lambs.

Chorioamnionitis is a frequent antecedent of preterm delivery with up to two thirds of infants born extremely preterm exposed to intrauterine inflammation. Preterm newborns exposed to intrauterine inflammation are at high risk of brain injury in the neonatal period (31, 32) and cerebral palsy later in childhood (11–13). This vulnerability is based on the involvement of inflammatory processes that disrupts brain architecture, induces neuronal apoptosis and increases cerebral metabolic demand (4, 17, 33). Moreover, antenatal inflammation leads to hemodynamic impairment, cerebral blood flow instability and loss of cerebral autoregulation (34). Lipopolysaccharide has been used extensively to model intrauterine inflammation in many species, as it produces a profound and consistent inflammatory response within the fetus, which accurately mimics that observed in humans (35). Our previous studies have shown that preterm lambs exposed to antenatal inflammation have high cerebral oxygen consumption and metabolic demand and a slower rate of increase in pulmonary blood flow and left ventricular output immediately after birth (5, 22), indicating an impaired cardiopulmonary transition. Further, ventilation-induced brain injury is exacerbated after 2 or 4 days exposure to antenatal LPS, with increased diffuse white matter injury and vascular extravasation (a marker of hemorrhage) (36). In the present study we showed that ICC after antenatal inflammation induces some of the detrimental hemodynamic effects already demonstrated in preterm lambs (9), including profound disturbance in cardiovascular function resulting in a rapid and transient increase in cerebral blood pressure and flow. These rapid fluctuations in cerebral pressure and flow are a known cause of cerebrovascular injury in preterm newborns (37, 38) and the injury is likely to be further increased if the infant is exposed to chorioamnionitis, which further impairs cerebral autoregulation (34). In contrast, the PBCC group maintained a stable hemodynamic transition throughout the study, particularly during the initiation of ventilation and cord clamping period. Thus it is likely that improving circulatory stability during the transition at birth by PBCC will be even more beneficial for infants exposed to chorioamnionitis.

The rapid fluctuations in blood pressure, flow and heart rate observed in ICC lambs upon cord clamping, and overshoot upon ventilation onset is consistent with that observed previously in preterm lambs (9). The cause of the cardiovascular instability is due to the removal of the placenta prior to aeration of the lung, resulting in an inability of the newborn to increase pulmonary blood flow and therefore establish the pulmonary circulation as the source of left ventricular preload previously provided by the placenta. We did not observe the subsequent rapid falls in heart rate and cerebral blood flow prior to ventilation in ICC lambs exposed to inflammation as we observed in the Bhatt study, as ventilation was initiated within 30 s after cord clamping. This largely mitigated worsening cardiovascular function secondary to progressive hypoxia as demonstrated previously (39). However, realistically in clinical practice it is unlikely that adequate respiratory support is rarely initiated within this short time (40) so the cardiovascular consequences of ICC after antenatal inflammation may be underestimated in our study. Importantly, blood pressure remained higher in ICC lambs than PBCC lambs for the first 20 min of the study. We previously demonstrated in asphyxiated near-term lambs that PBCC prevented the overshoot in blood pressure that occurs upon restoration of cardiac output after moderate asphyxia (41). Irrespective of the severity, the initial physiological response to perinatal hypoxia is identical and extremely well described. Initially it involves bradycardia, apnea (42, 43), a mild hypertension and an inhibition of body movements, along with a marked redistribution of cardiac output to increase blood flow and maintain oxygen supply to the heart and brain (44, 45). Further, there is a well-established peripheral chemoreceptor mediated rebound hypertension which persists for 2–3 min (46). When umbilical cord clamping occurs during fetal adaptation to hypoxia, the sudden reduction of cardiac output by 50% undermines the newborns capacity to adapt to the hypoxia and maintain oxygen delivery to the heart and brain, and sustains the rebound hypertension as observed in this study. PBCC completely mitigated the post-hypoxia hypertension and maintained a consistent heart rate throughout delivery. Given that asphyxia resuscitation and maternal chorioamnionitis are both independent risk factors for IVH in preterm infants (4, 47), PBCC may be more beneficial in these infants.

We previously demonstrated that PBCC clamping resulted in a smoother systemic and cerebral oxygenation in preterm lambs with ICC before ventilation onset resulting in rapid arterial and cerebral desaturation (10). Our data in this study confirms the deleterious effect of ICC before ventilation onset on cerebral and systemic oxygenation. In the ICC group we observed a dramatic decrease in SaO_2_, femoral StcO_2_, and cerebral StcO_2_ that lasted almost 2 min (Figure 1). In the study by Polglase et al., the mean time between umbilical cord clamping and ventilation onset in the ICC group was 79 s (10). In our study, although ventilation started only 30 s after umbilical cord clamping, the fall of arterial (~24%), cerebral (~10%), and peripheral (~11%) oxygenation was consistent and lasted for a significant period of 2–3 min. These findings are of concern given the known association between prolonged episodes of hypoxia and preterm brain injury (17) and considering that the time it takes to transfer the infant from the delivery table to the resuscitation area and to initiate adequate respiratory support is usually longer than 30 s (40). PBCC prevented this rapid reduction in oxygenation again highlighting the potential benefits of PBCC in clinical settings.

In our study, PBCC lambs had a higher AaDO_2_ and airway pressure requirements as well as lower tidal volumes and dynamic lung compliance than ICC lambs, indicating that respiratory function was reduced. A lower respiratory system compliance likely explains the requirement for higher pressures, which were limited to 40 cmH_2_O, and a lower target V_T_. While it is unclear why this occurred in PBCC lambs, as we would expect lung maturity to be identical between the groups, we consider this to be primarily a lung aeration issue. One explanation is that during PBCC, lambs were positioned laterally during the initiation of ventilation to avoid stretching the umbilical cord, whereas all ICC lambs were placed supine. As a result, the size of the dependent lung, particularly if placed on their right side, is much greater when lambs are placed laterally leading to reduced lung aeration. As humans have a longer umbilical cord and a different shaped chest, this is unlikely to be a problem in humans infants during DCC. Indeed clinical studies have not detected any differences in respiratory outcomes between DCC and ICC and, on the contrary, have shown better respiratory outcomes in preterm infants (48), although no study have isolated babies previously exposed to chorioamnionitis. Nevertheless, further investigations are warranted within this subgroup to ensure PBCC's safety in this subgroup of infants.

While our data support the hypothesis that the primary benefit of DCC is a more stable cardiovascular transition, our study is limited by a lack of blood volume measurements. However, there were no differences in hemoglobin levels between groups during the study period suggesting no significant transfusion occurred. In our study, lambs were delivered via cesarean section while the ewe was receiving ventilation under anesthesia. While this allowed us to demonstrate the importance of the timing of ventilation onset relative to umbilical cord clamping in a controlled situation, it does have limitations in transition to the delivery room of human infants. The absence of fetal respiratory effort due to maternal anesthesia, coupled with the absence of labor, makes the scenario somewhat different from most clinical scenarios. Indeed, the inter-relationship of other factors that can affect the cardiovascular transition, including labor or spontaneous breath efforts, were not assessed in the present study and require further studies to evaluate.

## Conclusion

PBCC in preterm lambs exposed to intrauterine inflammation resulted in a stable cardiovascular transition as well as maintaining systemic and cerebral oxygenation during transition compared to ICC lambs. PBCC also prevented the post-hypoxia overshoot in blood pressure observed in ICC lambs. However, PBCC lambs had worse respiratory mechanics resulting in higher ventilatory requirements which warrants further investigation in this sub-group of preterm neonates.

## Author contributions

AL and GP wrote manuscript and analyzed data. CA, VZ, TM, DL, SH, AG, MK, IN, GV, AL, and GP assisted with animal work. All authors revised the manuscript and contributed to data interpretation and revision of the manuscript. AL, GP, SH, and GV were responsible for study conception, design manuscript revision, and oversight of the research analyzed data.

### Conflict of interest statement

The authors declare that the research was conducted in the absence of any commercial or financial relationships that could be construed as a potential conflict of interest.
